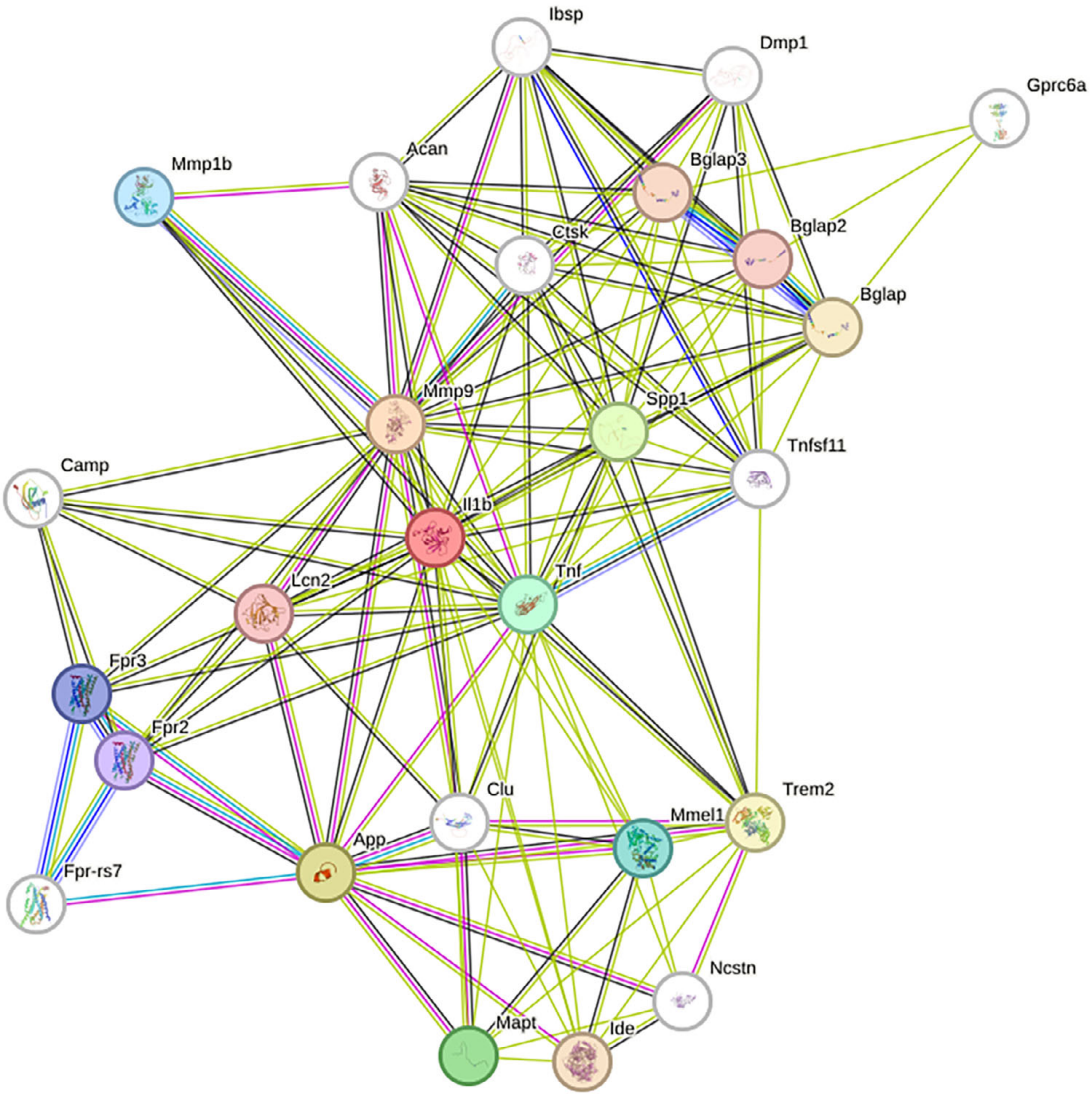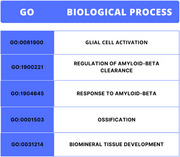# Protein‐Protein Interaction Analysis of Osteopontin (SPP1) in Alzheimer's Disease

**DOI:** 10.1002/alz70855_100614

**Published:** 2025-12-23

**Authors:** Luara Bela Rocha Gomes, Carlos Wagner Leal Cordeiro Júnior, Urias Silva Vasconcelos

**Affiliations:** ^1^ Faculty UNIRB Teresina, Teresina, Piauí, Brazil; ^2^ Laboratory of Neuroscience (LIM‐27), Faculty of Medicine, University of São Paulo, Brazil, Brazil, São Paulo, São Paulo, Brazil; ^3^ University of Sao Paulo (USP), São Paulo, São Paulo, Brazil; ^4^ Universidade Federal do Piauí, Teresina, PI, Brazil

## Abstract

**Background:**

Osteopontin (SPP1) is a multifunctional glycoprotein that plays a central role in inflammation and tissue remodeling, acting as a critical mediator in the progression of Alzheimer's disease (AD). It interacts with macrophages, microglia, and astrocytes, activating inflammatory signaling pathways that may worsen neurodegeneration through interactions with pathological proteins such as beta‐amyloid (APP) and tau (MAPT). Additionally, periodontal infections, which increase plasma levels of osteopontin, may amplify neuroinflammation, further accelerating AD progression. This study aims to investigate the molecular mechanisms linking osteopontin to AD progression, focusing on its interactions with pathological proteins and inflammatory pathways.

**Method:**

Protein interactions of SPP1 with AD‐associated proteins were mapped using the STRING platform, integrating experimental, gene co‐expression, and computational data. Targets analyzed included SPP1, APP, MAPT, IL1B, TNF, MMP9, and CXCL8, with a high confidence threshold (0.7). Interaction networks were visualized using Cytoscape, identifying central proteins (hubs), and Gene Ontology (GO) analysis highlighted relevant biological processes and pathways. Public data from GEO were used to assess SPP1 gene expression and its correlation with APP, MAPT, IL1B, and TNF in brain tissues.

**Result:**

Protein interaction analysis revealed that osteopontin (SPP1) interacts directly with APP, MAPT, IL1B, TNF, MMP9, and CXCL8, all central to inflammation and AD progression. SPP1 significantly interacts with APP and MAPT in neuroinflammation, mediated by IL1B and TNF. Osteopontin activates the NF‐κB pathway, implicated in chronic inflammation and neurodegeneration. The interaction with MMP9 suggests osteopontin may promote extracellular matrix remodeling, exacerbating neuronal damage. CXCL8 recruits inflammatory cells to the brain, facilitating neuroinflammation. Gene expression analysis showed elevated SPP1 in AD patient brain samples, correlating with APP (*p* < 0.05), MAPT (*p* < 0.01), and inflammatory cytokines IL1B (*p* < 0.001) and TNF (*p* < 0.001).

**Conclusion:**

SPP1 is crucial in AD, interacting with APP and MAPT, activating the NF‐κB pathway, amplifying neuroinflammation, and exacerbating neuronal damage. Elevated SPP1 expression correlates with increased APP, MAPT, and inflammatory cytokines. Periodontal infections that raise osteopontin levels may accelerate AD progression, suggesting modulation of osteopontin and control of periodontal inflammation as promising therapeutic strategies.